# Is the Peripheral Zone Thickness an Indicator of a Learning Curve in Bipolar Transurethral Plasma Enucleation of the Prostate?—A Single Center Cohort Study

**DOI:** 10.3389/fsurg.2021.795705

**Published:** 2022-02-02

**Authors:** Qihua Wang, Rami Alshayyah, Yi He, Lijie Wen, Yang Yu, Bo Yang

**Affiliations:** Third Department of Urology, The Second Affiliated Hospital of Dalian Medical University, Dalian, China

**Keywords:** AEEP, benign prostatic hyperplasia, peripheral zone, learning curve, bipolar

## Abstract

**Background:**

We conducted this cohort study to assess the differences in the learning curve of bipolar transurethral plasma enucleation of the prostate (B-TUEP) associated with prostatic peripheral zone thickness (PZT) under MRI quantitative measurements.

**Methods:**

For the study, 60 patients with benign prostatic hyperplasia (BPH) were involved. PZT are defined as “Thin” (<7 mm), “Thick” (>10 mm), and “Medium” (in between), with 20 patients in each group. Learning stages were defined as Group 1 (No. 1–20), Group 2 (No. 21–40), and Group 3 (No. 41–60). We measured parameters of the prostate, such as PZT and transitional zone thickness (TZT), with MRI. A learner with no experience in enucleation performed the operations. Statistical analyses were performed to compare the differences. Pearson correlation analysis and multiple linear regression analysis evaluated the relationship between characteristics of patients. *P* < 0.05 was deemed statistically significant.

**Results:**

One-Way ANOVA revealed different enucleation efficiency (0.811 ± 0.18 vs. 0.748 ± 0.14 vs. 0.634 ± 0.16), prostate volume (58.9 ± 15.33 vs. 57.3 ± 15.58 vs. 46.6 ± 14.10), and thickness of transition zone (44.45 ± 7.60 vs. 42.45 ± 6.08 vs. 34.78 ± 6.04) among Thin, Medium, and Thick groups. The enucleation efficiency is different between groups divided by learning stages (Group 1 vs. Group 3, 0.658 vs. 0.783; Group 2 vs. Group 3, 0.751 vs. 0.783). Pearson correlation analysis reveals that PZT was negatively correlated with prostate volume (*r* = −0.427), resection weight (*r* = −0.35), enucleation efficiency (*r* = −0.445), and TZT (*r* = −0.533), and was positively correlated with Q-max (*r* = 0.301) and bladder outlet obstruction index (BOOI) (*r* = 0.388). The regression coefficients of PZT, TZT, prostate volume, and Q-max were −0.012, 0.008, 0.007, and 0.013, respectively (all *P* < 0.05).

**Conclusion:**

Lower PZT is independent of higher enucleation efficiency, larger adenoma, and higher TZT. PZT may be an important factor on the learning curve of B-TUEP. Higher TZT, prostate volume. and Q-max may also relate to higher enucleation efficiency. For B-TUEP learners, it seems easier to perform the operation when the PZT is low, though more care should be taken with the capsule perforation. Further, the capsule plane should be maintained more attentively if the PZT is high.

## Introduction

Benign prostatic hyperplasia (BPH), as a disease which progresses with age, is one of the most important factors of lower urinary tract symptom (LUTS) in elderly men (1, 2). Anatomical endoscopic enucleation of the prostate (AEEP) is a popular technique for the surgical intervention of severe BPH, and has been widely accepted and recommended by many urologists (3). Less blood loss, shorter postoperative hematuria time, less diluted hyponatremia, and higher prostate adenoma removal rate are considered as significant advantages of this technique (4–6). At present, BPH is thought to be caused by hyperplasia of the transitional zone (7), which makes the adenoma of the transitional zone more circular under pressure (8). At the same time, the peripheral zone becomes thinner and forms the anatomical level of enucleation technology (9, 10). However, AEEP is considered to have a certain learning curve because of the requirement of higher operation skills (3, 11).

In order to help surgeons get through the learning curve more quickly, our center has run courses on bipolar plasma transurethral enucleation of prostate (B-TUEP) for urologists from other centers. We found that some characteristics may affect the efficiency of mastering this technique during teaching. On the other hand, our surgeons with enough experience in B-TUEP noticed that patients with thinner prostate peripheral zones seem to have a clearer capsule plane during AEEP procedure, which seems to be more beneficial to learners. Thus, we conducted a cohort study to assess the differences in the learning curve of B-TURP associated with different prostatic PZT, attempting to provide a theoretical basis for suitable patient selection.

## Patients and Methods

### Study Design

Sixty men diagnosed with BPH were designed to be included, and were numbered 1–60 according to the sequence of receiving operations. All of the patients were admitted because of severe LUTS, severe hematuria, bladder calculus formation, or refractory urinary retention. Exclusion criteria was a diagnosis or history of prostate cancer, prior prostate surgery, urethral strictures, and neurogenic bladder. We also excluded patients who had developed acute prostatitis within the last 6 months and whose prostate volumes were over 90 ml, because we considered that these situations would create serious difficulty for beginners. All the patients stopped their anticoagulation and antiplatelet medicine and received subcutaneous low-molecular heparin until 12 h before surgery, because their factors have been proven to increase hospital stay and complications (12). The preoperative evaluation of patients included serum prostate specific antigen (PSA), f-PSA, urine analysis, ultrasound, and prostatic multi-parameter 3.0MRI. Ultrasound-guided transrectal prostate biopsies were performed on patients suspected of prostate cancer due to elevated PSA, results of digital rectal examination, or imaging examination. Subjective and objective micturition parameters include preoperative international prostate symptom score (IPSS), quality of life score (Qol), Q-max, voiding detrusor pressure at Q-max, bladder outlet obstruction index (BOOI), and post-voiding residual volume (PVRV). Q-max, voiding detrusor pressure at Q-max, and BOOI were measured with urodynamic examination. PVRV was measured with ultrasound. All patients provided written informed consent. The study protocol was approved by the Institutional Review Board of our hospital in compliance with the Declaration of Helsinki. The length, width, and height of the prostate were measured by a radiologist using MRI, and the prostate volume was estimated by the widely accepted prostate volume calculation formula (prostate volume = length ^*^ width ^*^ height ^*^π/6). According to the description of Kwon et al. (1), peripheral zone thickness (PZT) is defined as the longest distance between the outer and inner edge of the peripheral zone when a straight line is drawn from the center of the transitional zone to the outer edge of the peripheral zone on the level of the largest transitional zone. Transition zone thickness (TZT) is the maximum straight distance between the two edges of the transition zone passing through the center. We measured the thickness bilaterally and took the average values as PZT and TZT, respectively.

We defined PZT <7 mm as “Thin,” over 10 mm as “Thick,” and the thickness between them as “Medium.” Patients were included in these groups according to different thicknesses, until we got 20 cases in each group. In addition, the No.1–20, 21–40, and 41–60 patients in the sequence of receiving surgery were divided into Group 1, Group 2, and Group 3, respectively, which represented different learning stages.

The enucleation time refers to time from the first incision to enucleation of the entire adenoma and complete hemostasis, which demands the condition of no active hemorrhage without fluid perfusion. According to Netsch et al. (13), the enucleation efficiency is defined as the resected weight/enucleation time (g/min). All surgeries were performed by one resident surgeon (surgeon A), who has completed more than 1 year's training in transurethral endoscopy in our hospital. However, he lacked experience in AEEP surgery, and has only performed resection and coagulation after enucleation performed by other surgeons in about 30 cases of B-TUEP. Surgeon B, who is the tutor of surgeon A, is experienced, and has performed more than 1,000 cases of B-TUEP. By watching surgeon B's operations and surgery videos, as well as discussing and consulting with surgeon B, surgeon A gained enough theoretical basis. Surgeon A had performed 10 B-TUEP operations with the help of surgeon B before. These 10 patients were excluded from the research.

The operation was performed with a bipolar plasma kinetic system, using 28Fr bipolar resectoscope and electrode loop. The PK system used 160W for cutting and 80W for coagulation. Normal saline was used as irrigation fluid. The operation was performed according to Liu et al.'s (14) report. Anatomical marks such as verumontanum, prostate, external urethral sphincter, and bilateral ureteral orifice were identified before beginning. A “λ”-shape incision was made close to the verumontanum to expose the surgical capsule. The adenoma was pushed from 6:00 position to both sides to form a transverse groove in the capsule plane. From 6:00 position in the groove, the adenoma was pried upward to the 12:00 direction while the plane was extended to both sides until the endoscope broke through at the bladder neck. The middle lobe was then nearly enucleated. The bilateral lobes of adenoma were then detached clockwise or counter-clockwise from 5:00 or 7:00 position of the apex until reaching the 12:00 position at bladder neck in a spiral manner. Then the adenoma was pushed toward the midline to separate adhesion from the bladder neck. After that, we could see the residual urethral mucosa connected to the prostatic apex at 12:00 position, which might contain parts of the external urethral sphincter. Then a horizontal incision was made as close as possible to the adenoma at the apex to keep more urethral mucosa. Then the capsule was fully enucleated except for a little fixation at 5:00 position, which was convenient for morcellation with electrode loop. During the whole operation, if bleeding or obvious blood vessels were seen, electrocoagulation was started to stop or prevent bleeding immediately. If the surgical field was not clear, it was first considered if there were bleeding points in or out of the visible field, or factors affecting irrigation. The resected tissue was fully flushed out and weighed. After meticulous hemostasis, a 20Fr three-lumen silicone catheter was indwelled.

### Statistical Analysis

Statistical analysis was performed using the statistical software program SPSS (IBM SPSS Statistics 25.0, Chicago, USA). P-P diagram, Q-Q diagram, histogram, and K-S test were used to identify normality of variables. Some variables with partial skewness distribution were transformed to normal distribution after natural logarithmic transformation. Continuous variables with normal distribution are described with mean ± standard deviation (SD). Differences in continuous variables between groups were analyzed via One-Way ANOVA. *Post-hoc* Multiple Comparisons were carried out by the LSD method. The variables with skew distribution are described with median (interquartile distance), whose comparison between groups and ordered variable are analyzed with Kruskal-Wallis H's test. Correlation analysis of continuous variables was performed by Pearson correlation analysis. A multiple linear regression analysis with stepwise selection of the variables was performed to quantify the relative contribution *R*^2^ of these parameters (IPP, sequence of receiving operation, PSA, f-PSA, PSAD, Q-max, PVRV, IPSS-T, IPSS-S, IPSS-V, Qol, voiding detrusor pressure at Q-max, BOOI, BMI, age, prostate volume, TZT, and PZT) to the variance of the dependent variable (enucleation efficiency). A stepwise approach was selected, integrating independent parameters sequentially. Collinearity was tested with the variance inflation factor (VIF). VIF > 5 indicated existence of collinearity. Durbin-Watson value close to 2 indicates that there is no autocorrelation in the model and no correlation between sample data. A significance level of *P* < 0.05 was deemed statistically significant.

## Results

**Figure 1:**
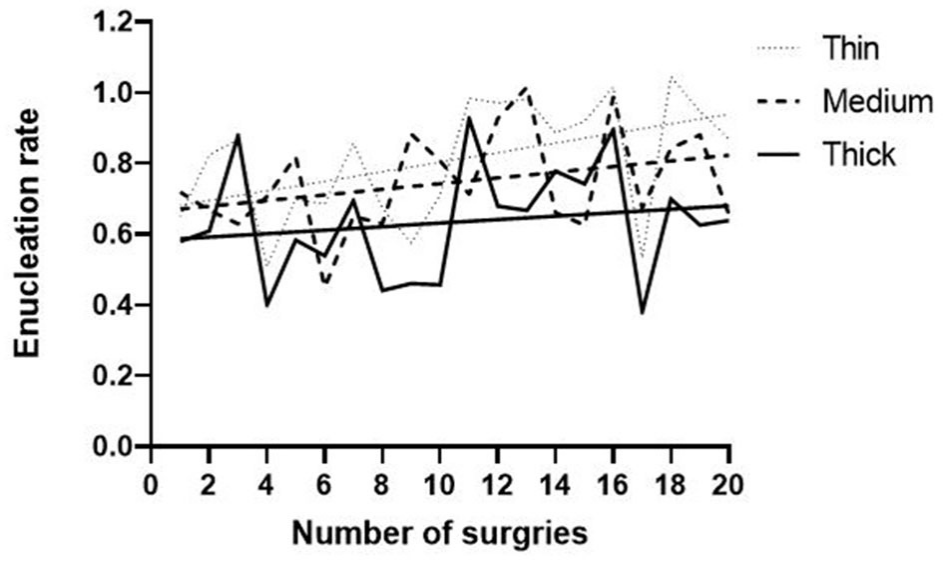
Learning curve of enucleation rate with different peripheral zones.

All sixty patients went through operations successfully, without conversion to open surgery or traditional transurethral resection of prostate (TURP) and perioperative death. None of the patients developed severe post-operation dilutional hyponatremia. Fifty-three patients had their catheters removed successfully 3 days after surgery, while the other seven patients developed urinary retention and were catheterized again until 1 week after operation. Twelve patients developed mild stress urinary incontinence for a short time (<1 month) after surgery; moreover, all of them developed no or little leakage on the 3-month follow-up. Two patients suffered from hematuria after discharge that may be due to constipation, and one patient got successful hemostasis after re-catheterization and continuous bladder irrigation. One patient developed bladder tamponade, which required blood clots evacuation and electrocoagulation to stop the bleeding, but no blood transfusion was required. The general trends of efficiency of enucleation are elevation with the gradual study, which is higher in the Thin group (Figure 1).

There is no difference in surgical sequence, age, height, body weight, BMI, PSA, f-PSA, PSAD, IPSS-S, IPSS-V, IPSS-T, Qol, PVR, Q-max, voiding detrusor pressure at Q-max, BOOI, enucleation time, or resection weight among the three groups. However, there are significant differences in efficiency, volume, and TZT of the three groups (Table 1). As a result, it is noticed that the thinner the PZT is, the higher the enucleation efficiency (0.811 ± 0.18 vs. 0.748 ± 0.14 vs. 0.634 ± 0.16, *P* = 0.004), the larger the prostate volume (58.9 ± 15.33 vs. 57.3 ± 15.58 vs. 46.6 ± 14.10, *P* = 0.025), and the thicker TZT are (44.45 ± 7.60 vs. 42.45 ± 6.08 vs. 34.78 ± 6.04, *P* < 0.001). In *Post-hoc* Multiple Comparisons (Supplementary Material 1), there are differences in resected tissue weight (50.0 ± 14.56 vs. 39.2 ± 13.48, *P* = 0.018), PSAD [0.091 (0.085) vs. 0.144 (0.168), *P* = 0.039], and age (72.6 ± 5.63 vs. 68.5 ± 5.39, *P* = 0.025) between the Thin group and Thick group.

**Table 1:** Clinical data of patients in different PZT groups.

	**Thin (*n =* 20)**	**Medium (*n =* 20)**	**Thick (*n =* 20)**	***F*-value**	***P*-value**
Surgical sequence	34 ± 16.74	25 ± 15.68	32 ± 19.22	1.643	0.202
Age (y)	71.0 ± 5.86	72.6 ± 5.63	68.5 ± 5.39	2.691	0.076
Weight (kg)	73.2 ± 12.51	70.3 ± 10.76	71.1 ± 8.47	0.377	0.688
Height (cm)	170.6 ± 5.38	171.9 ± 5.01	172.3 ± 4.42	0.645	0.529
BMI (kg/m^2^)	25.1 ± 4.09	23.9 ± 3.97	23.9 ± 2.22	0.854	0.431
Prostate volume (ml)	58.9 ± 15.33	57.3 ± 15.58	46.6 ± 14.10	3.942	0.025
IPP	7.46 ± 8.72	7.82 ± 8.70	8.88 ± 10.12	0.128	0.880
^*^PSA (ng/dl)	4.935 (5.980)	4.785 (7.702)	5.715 (8.493)	0.637	0.533
^*^f-PSA (ng/dl)	0.980 (1.108)	0.860 (1.001)	1.130 (0.940)	0.170	0.844
^*^PSAD	0.091 (0.085)	0.099 (0.077)	0.144 (0.168)	2.675	0.078
Q-max (ml/s)	2.8 (4.4)	1.9 (5.2)	3.9 (6.0)		0.561
Voiding detrusor pressure at Q-max (cm H_2_O)	65.4 ± 27.47	84.4 ± 34.37	82.5 ± 32.32	1.649	0.204
BOOI	67.6 ± 23.27	74.0 ± 39.08	87.9 ± 31.92	1.167	0.324
PVRV (ml)	112.5 (223.7)	44.5 (150.3)	96 (395)		0.598
IPSS-T	29 (11.2)	33.5 (10.5)	31 (7.7)		0.325
IPSS-S	12 (4.7)	15.0 (4.7)	13.5 (4.0)		0.394
IPSS-V	16.5 (8.7)	18.5 (6.7)	19 (7.2)		0.562
Qol	5 (1.0)	5 (2.0)	5 (1.7)		0.971
TZT (mm)	44.45 ± 7.60	42.45 ± 6.08	34.78 ± 6.04	11.929	<0.001
PZT (mm)	5.67 ± 1.26	8.27 ± 0.71	11.98 ± 2.34	79.791	<0.001
Tissue weight (g)	50.0 ± 14.56	46.3 ± 14.07	39.2 ± 13.48	3.085	0.053
Enucleation time (min)	61.0 ± 8.55	60.8 ± 10.23	61.0 ± 10.01	0.002	0.998
Enucleation efficiency (g/min)	0.811 ± 0.18	0.748 ± 0.14	0.634 ± 0.16	6.040	0.004

*Continuous variables with normal distribution are represented by mean ± SD, while ones with skewed distribution are represented by median (interquartile distance).*

*The variables are tested for homogeneity of variance, and then the F-value was calculated by One-way ANOVA test. Ordered variables are analyzed with Kruskal-Wallis H's test.*

*P < 0.05 indicated significant statistical significance.*

* *The variables present normal distribution after natural logarithmic transformation and F and P-value are calculated according to the transformed data*.

There is no significant difference in the above parameters among the three groups divided by the learning progress (Supplementary Material 2). In fact, the enucleation efficiency of Group 3 is significantly higher than that of Group 1 (0.783 vs. 0.658, *P* = 0.024) and Group 2 (0.783 vs. 0.751, *P* = 0.031) in the *Post-hoc* Multiple Comparisons (Supplementary Material 3).

In addition, Pearson correlation analysis (Table 2) gives the result that PZT was negatively correlated with prostate volume (*r* = −0.427), resection weight (*r* = −0.35), and enucleation efficiency (*r* = −0.445) in moderate intensity, and strongly negatively correlated with TZT (*r* = −0.533). On the contrary, it was moderately positively correlated with Q-max (*r* = 0.301) and BOOI (*r* = 0.388), which were statistically significant.

**Table 2:** Pearson correlation analysis of PZT.

	***r***	***P*-value**
Prostate volume (ml)	−0.427	0.001
Tissue weight (g)	−0.35	0.006
Enucleation efficiency (g/min)	−0.445	<0.001
TZT	−0.533	<0.001
BOOI	0.388	0.021
^*^PSA	−0.004	0.976
^*^f-PSA	0.029	0.824
^*^PSAD	0.239	0.066

*r: correlation coefficient.*

*P <0.05 indicates statistical significance.*

* *Fits normal distribution after natural logarithmic transformation*.

We also conducted multiple linear regression analysis. As can be seen from Table 3, TZT, PZT, prostate volume, and Q-max are finally included in the model as independent variables affecting the enucleation efficiency. The model formula is that enucleation efficiency = 0.041 + 0.008 ^*^ TZT − 0.012 ^*^ PZT + 0.007 ^*^ volume + 0.013 ^*^ Q-max (*R*^2^= 0.932). This means that TZT, PZT, prostate volume, and Q-max can explain the 93.2% change of enucleation efficiency, and the model fitting degree is high. The F test (*F* = 48.050, *P* < 0.001) shows that at least one of these variables has a significant influence on the enucleation efficiency. In addition, the VIF values in the model are all less than five, which means that there is no collinearity among independent variables. Moreover, the D-W value is very close to 2, which shows that there is no autocorrelation in the model and correlation between the sample data. The regression coefficients of TZT, prostate volume, and Q-max were 0.008 (*t* = 3.597, *P* = 0.001), 0.007 (*t* = 7.275, *P* < 0.001), and 0.013 (*t* = 2.538, *P* = 0.017), respectively. The regression coefficient of PZT is −0.012 (*t* = −2.080, *P* = 0.046). According to the analysis, TZT, prostate volume, and Q-max have a significant positive influence on the gouging efficiency, while PZT has a significant negative influence.

**Table 3:** Multiple linear regression analysis.

	**Unstandardized**		**Standardized coefficients**	***t***	***P***	**VIF**	***R* ^2^**	**Adjusted *R*^2^**	***F***
	**coefficients**								
	**B**	**Std. Error**	**Beta**						
Constant	0.041	0.101	–	0.410	0.685	–	0.932	0.851	*F* = 48.050, *P* < 0.001
TZT	0.008	0.002	0.334	3.597	0.001^**^	1.907			
PZT	−0.012	0.006	−0.150	−2.080	0.046^*^	1.150			
Prostate Volume	0.007	0.001	0.646	7.275	<0.001^**^	1.746			
Q-max	0.013	0.005	0.173	2.538	0.017^*^	1.026			

*Dependent Variable: Enucleation Efficiency.*

*Durbin-Watson: 2.136.*

* *P < 0.05,*

** *P < 0.01*.

## Discussion

B-TUEP has been recognized to provide symptom relief for BPH patients with prostates of almost any size, with definite efficacy and safety (15). Moreover, some researchers have confirmed that the incidence of body pain/discomfort, decreased physical pleasure, and difficulty in ejaculation of patients after B-TUEP is lower than that of holmium laser enucleation of prostate (HoLEP) (16). The overall enucleation efficiency in our study is similar to previous published articles about learning curves of different AEEP techniques (13, 17, 18). Clinically we found that some special characteristics of prostates are not suitable for doctors who have just started to learn enucleation technology, as they may affect their confidence in mastering this technology and limit its wide application. It is helpful for them to start with easier cases and be aware of complications that are more likely to occur in different PZT groups.

In our study, we firstly divided the patients into groups according to PZT. It was revealed that the enucleation efficiency of adenoma with PZT <7 mm was significantly higher than that of adenoma with PZT over 10 mm (Figure 1). This indicated that lower PZT may be more adequate for beginners' learning curves in the aspect of enucleation efficiency. Moreover, it is interesting that the enucleation efficiency of the Medium group is obviously higher than that of the Thick group, while there is no difference in resected tissue weight between the two groups in *Post-hoc* Multiple Comparisons (Supplementary Material 1). This proves that PZT may be an important factor for enucleation efficiency.

**Figure 2:**
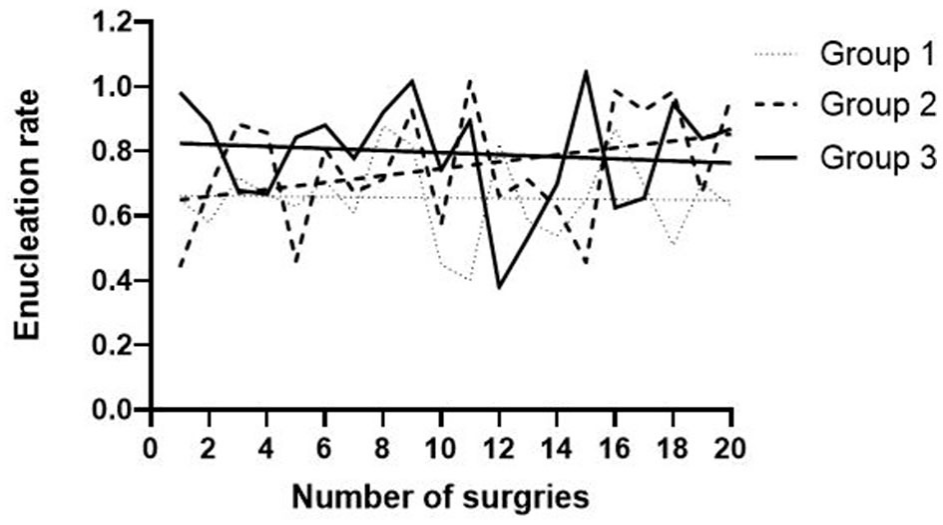
Learning curve of enucleation rate in different learning stages.

In the comparison of groups according to learning stages, the enucleation efficiency in the early stage is similar to that of middle stage, and obviously increases at the late stage (Figure 2). That indicates that enucleation efficiency is supposed to be a quantitative indicator of mastery of B-TUEP technique.

Our conclusion was further confirmed by multiple linear regression analysis. We found that when all the factors mentioned above were analyzed in stepwise approach, only TZT, PZT, prostate volume, and Q-max were proved to have the most significant influence on enucleation efficiency, which could explain 93.2% of enucleation efficiency changes. Moreover, the above variables do not display collinearity, suggesting that they are all independent factors, in which the increase of TZT, prostate volume, and Q-max can increase the enucleation efficiency, while the increase of PZT can decrease it. Our conclusion is in accordance with the work of Carolina Bebi et al. in which they found a larger prostate provided better enucleation efficiency.

It is worth noting that thin PZT seem to be more prone to developing capsule perforation during operation (Thin vs. Medium vs. Thick, 30 vs. 10 vs. 15%) (Table 4). We speculate that the peripheral zone is too thin to maintain the pressure during AEEP. When giving courses, we found that beginners' manual strength control is unstable and inexperienced. Especially when learners forget to change the direction of pushing, it will lead to the increase of pressure on the capsule. In this way, thinner capsules are more likely to be perforated, while thicker capsules may give them more opportunities to make mistakes. Well-defined planes greatly affect the development of perforation. However, more plane losses occurred in Group 3 than Group 1 and 2, which indicated that low PZT may be a greater risk factor for perforation. On the other hand, adenoma with high PZT seems to be more likely to develop multiple layers of capsule plane during enucleation (Thin vs. Medium vs. Thick, 20 vs. 15 vs. 40%), interfering with enucleation progress. However, Chi-square test cannot be performed due to the small sample size, so this conclusion needs to be confirmed by a larger sample study.

**Table 4:** Intraoperative and postoperative complications.

	**Clavien-Dindo group**	**Group 1 (*n =* 20)**	**Group 2 (*n =* 20)**	**Group 3 (*n =* 20)**
**Intraoperative complications**				
Wrong capsule plane	–	4 (20%)	3 (15%)	8 (40%)
Capsule perforation	I	6 (30%)	2 (10%)	3 (15%)
**Postoperative complications**				
Continuous hematuria	I	–	1 (5%)	–
Hematuria requiring surgically intervention	IIIa	1 (5%)	–	–
Hematuria requiring blood transfusion	II	–	–	–
Acute urinary retention after removal of the urethral catheter	I	1 (5%)	2 (10%)	4 (20%)
Short term stress urinary incontinence after removal of the urethral catheter	I	4 (20%)	5 (25%)	3 (15%)

Regarding the PZT, a previous study showed no tendency of thickening with age (7). It is also a meaningful anatomical factor and is related to prostate volume and micturition parameters (1). As BPH develops, the proliferation of the transitional zone has compression effects on urethra, peripheral zone, bladder, and surrounding tissue (1), thereby causing urinary tract obstruction and thinning of peripheral zone and intravesical prostatic protrusion (IPP). However, the compression effect on the peripheral zone could be low when the prostate volume is small, resulting in the lack of a well-defined mature layer formation that is crucial to identify surgical plane during enucleation (19). The result of our study indicates PZT may become thinner under pressure with the increase of age and the aggravation of BPH, due to the fact that patients in the Medium group are older than those in the Thick group. In our study, PZT was negatively correlated with prostate volume, tissue weight and TZT, and was positively correlated with Q-max. Therefore, our research is in common with the conclusion of Kwon et al. (1), that is, PZT of BPH patients may reflect the hyperplasia degree of prostate and the pressure on the peripheral zone, which may reveal the extent of urethral obstruction and voiding symptoms.

## Conclusion

Measurement of PZT under MRI is related to the learning curve of B-TUEP, so it can provide information for learners in selecting adequate patients pre-operationally. Better patient selection can help learners to go through the curve of B-TUEP faster. Prostates with lower PZT tend to be related to higher enucleation efficiency, larger adenoma, and higher TZT. In addition, higher TZT, prostate volume, and Q-max may be independently related to higher enucleation efficiency as well. For B-TUEP learners, the operation seems easier to perform when the PZT of prostate is low, though more care should be given with the capsule perforation. The capsule plane should be maintained more attentively if the PZT is high. Surgeons can estimate the difficulty of operation by measuring PZT before operation, so as to select more suitable patients at the beginning.

### Limitation

We divided patients into groups due to different PZT experimentally, because the cut-off points were never described in previous studies. Stratification of PZT may be improved by further studies. However, our results proved that PZT had an effect on the learning curve of B-TUEP. Our study was based on the learning process of Surgeon A, which may lack representativeness. We noticed that there are also some published studies that involved just a single surgeon (17, 19), and we consider it is valuable to share this experience with more surgeons who want to learn B-TUEP.

We did not describe postoperative parameters including length of hospital stay and postoperative irrigation. This is because we plan to discharge most patients 3 days after operation if they do not develop severe hematuria or other severe complications. Some patients still needed indwelling catheters when they left our hospital, and required moving to local hospitals and clinics.

## Data Availability Statement

The original contributions presented in the study are included in the article/Supplementary Material, further inquiries can be directed to the corresponding author/s.

## Ethics Statement

The studies involving human participants were reviewed and approved by Ethics Committee of the Second Affiliated Hospital of Dalian Medical University. The patients/participants provided their written informed consent to participate in this study. Written informed consent was obtained from the individual(s) for the publication of any potentially identifiable images or data included in this article.

## Author Contributions

QW is responsible for collecting data and writing articles. RA is responsible for reviewing articles and proofreading English. YH is responsible for the conception and statistical analysis of the article. LW is responsible for the revision of manuscripts. YY is responsible for the revision of manuscripts and interpretation of statistical analysis results. BY is the tutor of QW and RA and the surgical instructor in the article. All authors contributed to the article and approved the submitted version.

## Conflict of Interest

The authors declare that the research was conducted in the absence of any commercial or financial relationships that could be construed as a potential conflict of interest.

## Publisher's Note

All claims expressed in this article are solely those of the authors and do not necessarily represent those of their affiliated organizations, or those of the publisher, the editors and the reviewers. Any product that may be evaluated in this article, or claim that may be made by its manufacturer, is not guaranteed or endorsed by the publisher.

## Abbreviations

AEEP: Anatomical endoscopic enucleation of prostate
BPH: Benign prostatic hyperplasia
B-TUEP: Bipolar transurethral plasma enucleation of the prostate
MRI: Magnetic resonance imaging
PSA: Prostate specific antigen
f-PSA: free prostatic specific antigen
PASD: Prostate specific antigen density
IPSS: International Prostatic Symptom Score
Qol: Quality of life
BOOI: Bladder outlet obstruction index
PVRV: Post-voiding residual volume
PZT: Peripheral zone thickness
TZT: Transition zone thickness.
